# RT 3D TEE: Characteristics of Mitral Valve in Ischemic Mitral Regurgitation Evaluated by MVQ Program

**DOI:** 10.4021/cr63w

**Published:** 2011-07-25

**Authors:** Sylva Kovalova, Josef Necas

**Affiliations:** a Centre of Cardiovascular Surgery and Transplantation Brno, Czech Republic

**Keywords:** Ischemic mitral regurgitation, RT-3D echocardiography, Mitral Valve Quantification program, LV remodeling

## Abstract

**Aim:**

To assess the changes of mitral valve (MV) in ischemic mitral regurgitation (IMR) using Mitral Valve Quantification (MVQ) program.

**Methods:**

We examined 46 patients (18 women) with IMR aged 45-86 and a control group of 33 healthy individuals (14 women) aged 18-88. Following parameters were assessed: Area of minimal surface spanning annulus (A3), annulus height (h), tenting height (Th), exposed area of anterior (AL), posterior (PL) and both leaflets (BL), ejection fraction of the left ventricle (LV EF), regurgitation volume (RV) and BL/A3, AL/A3, PL/A3 ratios. The normal range of BL/A3 ratio was defined as the average ± 2SD of control group. The study group was separated into subgroup 1 with BL/A3 ratio within normal values and subgroup 2 with pathological BL/A3 ratio. Corresponding parameters of IMR group were compared to the controls and both subgroups were compared to each other using Student t-test.

**Results:**

In IMR group, as compared to the controls, A3, AL, PL, BL as well as BL/A3, AL/A3, PL/A3 ratios and Th were significantly increased, conversely, h and LV EF was significantly decreased. In the subgroup 2 as compared to the subgroup 1 there was significant increase of Th, BL, AL and PL, while EF LV was significantly decreased. There was no significant difference between these subgroups in A3, h and RV.

**Conclusion:**

In ischemic MV remodeling two stages were identified without relation to the severity of IMR. The first stage was mainly influenced by the LV dilatation while LV remodeling was more important in the second stage.

## Introduction

**Figure 1:**
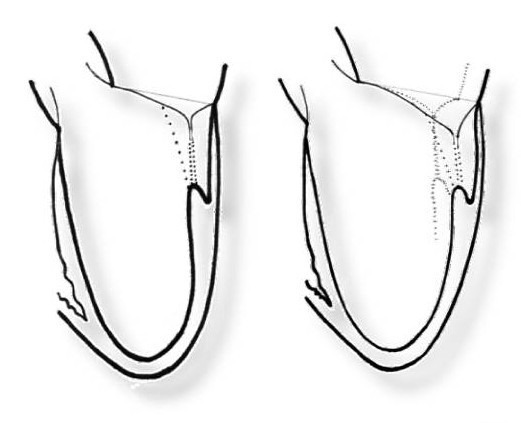
Mitral valve remodelling in ischemic heart disease.

The main feature of mitral regurgitation in ischemic heart disease (IHD) is its functional nature without structural abnormalities of the mitral valve. The underlying mechanism of ischemic mitral regurgitation is remodeling of the left ventricle (LV) and of the mitral valve itself as a part of the ventricle [1]. The remodeling process is started by the reduction of number and/or function of contractile elements of myocardium and depressed left ventricular function. Further,it is characterized by left ventricular dilatation including mitral annulus, increased LV sphericity followed by abnormal position of papillary muscles in relation to the mitral leaflets (Fig. 1). The consequential phases of the left ventricular remodeling comprise restrictive movement of the mitral leaflets (especially the posterior one), increase of mitral valve tenting (the point of coaptation is shifted deeper into the left ventricular cavity) and decrease of coaptation length resulting in reduced coaptation area.

RT 3D echocardiography is a new modality capable of exact assessment of mitral valve [2]. Mitral valve quantification (MVQ) program, which is a part of commercial software, enables to quantify many linear and nonlinear distances, nonplanar areas and atypical volumes. It provides completely new characteristics of mitral valve based on, so far, unavailable information.

The aim of the study was to assess the changes of mitral annulus and leaflets in ischemic mitral regurgitation.

## Methods

We examined 46 consecutive patients (18 women) referred for heart surgery with IHD and ischemic mitral regurgitation. They were aged 45-86 years (67 average). These patients had no structural abnormalities of the mitral valve; instead the regurgitation was due to the remodelling of the left ventricle with subsequent dilatation of the mitral annulus and changes in leaflets motion and coaptation.

We also investigated a control group of 33 healthy individuals (14 women) aged 18-88 years (56 average). These healthy controls were referred for TEE to exclude patent foramen ovale because of previous embolization to the central nervous system. The normal controls had no valve defect, no ventricular hypertrophy, and their left ventricular function was within normal limits. All were in sinus rhythm.

Transesophageal echocardiography was performed in all study subjects using 3D matrix transducer (X7-2T) on Philips iE33 system (Philips Medical Systems, Andover, MA, USA). Both live (zoom) and full volume acquisition were performed in all individuals. Full volume data were preferred for MVQ model construction because of wider angle of acquisition and higher frame rate in this mode. In patients with atrial fibrillation, live data were used for analysis. End-systolic model of mitral valve was created using MVQ program (version 7.1) within commercially available Q-lab software (Philips). The basis for the model construction is the 3D data set. In a series of 2D sections obtained from the 3D data the echocardiographer defines the annulus and commissures and traces the leaflets. The MVQ program then generates the valve model. A number of parameters of the mitral valve can then be quantified according to the selected preset.

**Figure 2:**
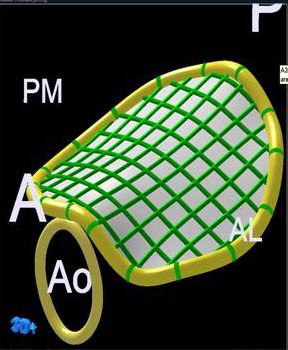
Area of minimal surface spanning annulus.

**Figure 3:**
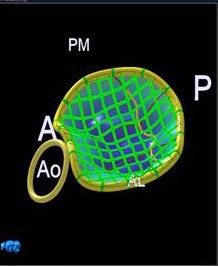
Exposed area of both leaflets.

We assessed following parameters:

1. Parameters defining mitral annulus: Area of minimal surface spanning annulus (A3) (Fig. 2), mitral annulus height (h).

2. Parameters expressing the relation between the annulus and the leaflets: Tenting height (Th), exposed area of both leaflets (BL) (Fig. 3), exposed area of anterior leaflet (AL), exposed area of posterior leaflet (PL) and BL/A3, AL/A3, PL/A3 ratios.

3. Other parameters: Ejection fraction (EF) of LV using Teichholz formula (2D) and regurgitation volume (RV) using hemiellipsoidal PISA method.

Corresponding parameters of patients with IHD were compared to healthy controls using Student t-test.

The normal range of BL/A3 ratio was defined as the average ± 2SD of control group. According to the BL/A3 value the study group was divided into 2 subgroups: In patients of subgroup 1 the BL/A3 ratio was within normal values while in patients of subgroup 2, BL/A3 ratio was higher than normal values. Corresponding parameters of both subgroups were compared to each other and to the control group as well, using Student t-test.

## Results

Control group of 33 healthy individuals (14 women) aged 18-88 years (average 56) had an average ejection fraction of 58.2%. All individuals were in sinus rhythm. Six were treated for hypertension but were normotensive following treatment. In the group with ischemic heart disease there were 46 study subjects (18 women) aged 45-86 years (average 67) with average ejection fraction of 39.8%. Thirty three (71.7%) were treated for hypertension and 12 (26.1%) had atrial fibrillation.

The measured parameters of the mitral valve in patients with IHD and healthy controls (A3, h, Th, AL, PL, BL, BL/A3, AL/A3, PL/A3 ratios, EF of LV) expressed as the average ± 2SD are shown in the table 1.

**Table 1:** Followed Parameters in IHD and Controls

	A3 (mm^2^)	h (mm)	h_t_ (mm)	BL (mm^2^)
IHD	1191.4 ± 396.4*	3.9 ± 2.2*	11.4 ± 5.0*	1438.0 ± 513.4*
Controls	955.8 ± 516.2	4.3 ± 1.4	5.9 ± 2.8	1046.3 ± 432.2

	AL (mm^2^)	PL (mm^2^)	BL/A3 (x100)	LV EF (%)
IHD	828.3 ± 325.6*	591.8 ± 264.2*	121.1 ± 14.6*	39.8 ± 21.6*
Controls	588.1 ± 239.2	459.1 ± 217.2	112.8 ± 7.34	58.2 ± 11.2

*indicate statistical significance (Student t-test) compared to controls.

In patients with ischemic heart disease, as compared to healthy individuals, LV EF was significantly depressed, area of minimal surface spanning annulus, exposed areas of leaflets as well as BL/A3, AL/A3, PL/A3 ratios were significantly increased. Mitral annulus height was lower in ischemic mitral regurgitant lesions and conversely, tenting height was higher than in healthy controls.

Group of patients with IHD was divided into two subgroups according to BL/A3 ratio. In patients of subgroup 1 in whom the BL/A3 ratio was within normal values, there were 19 individuals (7 women). In patients of subgroup 2, BL/A3 ratio was higher than normal values and this subgroup comprised 27 study subjects (11 women). Followed parameters of both subgroups, compared to the controls, are shown in the table 2.

**Table 2:** Subgroups of IHD Compared to the Controls

	A3 (mm^2^)	h (mm)	h_t_ (mm)	BL (mm^2^)
IHD BL/A3 normal	1146.1 ± 356.1*	4.0 ± 2.4	10.5 ± 5.0*	1312.8 ± 435.0*
IHD BL/A3 pathol.	1223.3 ± 410.4*	3.8 ± 1.8*	12.2 ± 4.0*	1526.1 ± 450.6*
Controls	955.8 ± 516.2	4.3 ± 1.4	5.9 ± 2.8	1046.3 ± 432.2

	AL (mm^2^)	PL (mm^2^)	LV EF (%)	
IHD BL/A3 normal	746.1 ± 242.0*	566.6 ± 229.8*	43.0 ± 22.1*	
IHD BL/A3 pathol.	886.1 ± 326.0*	639.8 ± 220.0*	37.6 ± 19.8*	
Controls	588.1 ± 239.2	459.1 ± 217.2	58.2 ± 11.2	

*indicate statistical significance (Student t-test) compared to controls.

Both subgroups of patients with ischemic mitral regurgitation as compared to healthy individuals had significantly larger area of mitral annulus as well as exposed areas of both leaflets. Tenting height in both subgroups of IHD was higher and conversely LV EF was lower than in normal individuals. Mitral annulus height was significantly decreased only in subgroup 2 while in subgroup 1 this parameter did not significantly differ from controls.

The comparison of followed parameters of both ischemic subgroups to each other can be seen in the table 3.

**Table 3:** Subgroups of IHD Compared to Each Other

	A3 (mm^2^)	h (mm)	h_t_ (mm)	BL (mm^2^)
IHD BL/A3 normal	1146.1 ± 356.1	4.0 ± 2.4	10.5 ± 5.0	1312.8 ± 435.0
IHD BL/A3 pathol.	1223.3 ± 410.4	3.8 ± 1.8	12.2 ± 4.0*	1526.1 ± 450.6*

	AL (mm^2^)	PL (mm^2^)	LV EF (%)	RV
IHD BL/A3 normal	746.1 ± 242.0	566.6 ± 229.8	43.0 ± 22.1	24.1 ± 20.0
IHD BL/A3 pathol.	886.1 ± 326.0*	639.8 ± 220.0*	37.6 ± 19.8*	24.7 ± 19.8

*indicate statistical significance (Student t-test).

In the subgroup of patients with pathological BL/A3 ratio, there was significant increase of tenting height and exposed areas of both leaflets, whereas their LV EF was significantly decreased. There was no significant difference between the ischemic subgroups in the annulus height and area of mitral annulus and in regurgitant volume.

## Discussion

Ischemic mitral regurgitation still remains a challenge-both its assessment and decision making concerning adequate heart surgery. Some pathophysiological mechanisms contributing to the ischemic mitral regurgitation are well described in the literature. Remodeling of the left ventricle together with its dilatation leads to atypical position of papillary muscles in relation to the mitral leaflets. Restrictive movement of the leaflets with increase of tenting height and the decrease of coaptation length and area are the consequences of the remodeling process [1]. 2D echocardiography provides only limited spectrum of measurements of mitral valve. Using 2D modality some parameters cannot be quantified at all, in some others, because of lack of spatial navigation, the accuracy and reproducibility of measurements cannot be guaranteed [3]. Conversely, RT 3D echocardiography with MVQ program ensures both the selection of exact site of measuring and moreover, it enables quantification of nonlinear and nonplanar structures not available so far.

In our study we evaluated mitral valve as a complex consisting of mitral annulus and leaflets. We analyzed both components separately and also the relations between them. In 2D echocardiography tenting height is generally accepted parameter reflecting the stage of LV remodelling. Area of minimal surface spanning annulus represents the real three-dimensional (nonplanar) area of mitral annulus. The value of this parameter, as well as the value of mitral annulus height, is mainly influenced by dilatation of the left ventricle (as mitral annulus is a part of the LV). The exposed area of both mitral leaflets is the part of the valve visible from the left atrium. Mathematically it can be defined as the area of the mitral valve with exclusion of the area of coaptation. Its size is influenced by the dilatation of mitral annulus, but also by abnormally deeper position of the leaflets due to the atypical position of papillary muscles in IHD. It changes also relations between closing and tethering forces of mitral valve. For this reason the coaptation area of mitral leaflets in IHD is decreased whereas the exposed area of leaflets increases. Evaluation of LV EF and regurgitation volume in our study represent assessment of consequences of functional changes of the mitral valve.

In our study group we found in patients with ischemic mitral regurgitation flattening of the annulus (decrease of mitral annulus height) and increase of the mitral annulus area (area of minimal surface spanning annulus) together with the increase of tenting height. These findings correlated well with the data in the literature [1, 4-9]and we described these changes of mitral annulus in our previous study as well [10]. On the contrary, the normal range of exposed area of mitral leaflets hasńt been published yet. It is a new, nonplanar parameter which was not available before the introduction of three-dimensional quantification. In our study we intentionally quantified also the BL/A3 ratio. We presumed that two components influence the remodeling of the mitral valve in IHD-simple dilatation of the left ventricle and other “remodeling factors” (position of papillary muscles, chordae etc). BL/A3 ratio may help to express the proportionality of these two components.

Among the patients with IHD two subgroups were differentiated according to the BL/A3 ratio-those with normal value (subgroup 1) and those with pathological value of this parameter (subgroup 2). All followed parameters with exception of the annulus height in both subgroups differed significantly from the healthy controls (Table 2). As compared to each other the subgroup 2 significantly differed from the subgroup 1 only in EF and parameters related to the leaflets (tenting height and exposed areas of both leaflets) (Table 3). There was no significant difference in the parameters of mitral annulus (its area and height). Based on these findings we consider these two subgroups to represent two phases of the remodeling process. The first phase is characterized mainly by the changes of mitral annulus-its dilatation and flattening. The increase of exposed area of leaflets is proportional to the annulus dilatation, BL/A3 ratio is within normal range. In the next phase on the contrary there is no significant progress of changes of the mitral annulus (neither dilatation nor flattening) and the significant increase of the exposed area of leaflets and tenting height can be explained by progressive remodelling of the LV.

As for the functional consequences of the remodelling process, there was no significant difference between the subgroups in regurgitation volume at rest. In literature the correlation of severity of ischemic mitral regurgitation with dilatation of the LV but not with LV EF is described, but all authors admit the effect of LV remodelling too [11-14]. However, we must emphasize that our study comprised only patients with significant ischemic mitral regurgitation referred to the valve surgery according to the contemporary guidelines. Patients with mild regurgitation referred to revascularization only without the valve surgery, were excluded.

In the future the evaluation of the stage of mitral valve remodelling may be useful in decision making about the valve surgery. It will be necessary to enlarge the study group, to assess the changes of RV in both subgroups during physical stress or to define some new parameters of mitral valve with good correlation to the regurgitant volume or treatment outcome. In this respect especially the evaluation of changes of subvalvular portion of the LV (chordae and papillary muscles) may help in decision making in mitral valve surgery [15].

### Conclusion

RT 3D echocardiography with MVQ program provides new parameters for assessment of the mitral valve in IHD. Using these parameters in patients with ischemic mitral regurgitation two stages of remodelling process of the LV (and mitral valve) can be identified. The changes of the mitral valve in the first stage are caused mainly by LV dilatation, while the LV remodelling is responsible for the second stage. The severity of ischemic mitral regurgitation doesn’t simply correlate with the remodelling stage.

### Limitations of the study

The main limitation is relatively small study group. A larger number of study subjects (both patients with IHD and healthy controls too) will refine the values of assessed parameters.

Patients were examined only at rest, the potential changes of the regurgitation volume during physical stress was not assessed.

Patients with mild (insignificant) ischemic mitral regurgitation were not included into the study, the range of regurgitant volume was relatively narrow.

Due to the limited resolution of RT 3D TEE (especially in the far field), the subvalvar apparatus of the mitral valve was not assessed.
